# Alternative Splicing and Tumor Progression

**DOI:** 10.2174/138920208786847971

**Published:** 2008-12

**Authors:** Claudia Ghigna, Cristina Valacca, Giuseppe Biamonti

**Affiliations:** Istituto di Genetica Molecolare – Consiglio Nazionale delle Ricerche, Via Abbiategrasso 207. 27100 Pavia, Italy

**Keywords:** Alternative splicing, cancer, EMT, splicing correction, splicing factors, biomarkers.

## Abstract

Alternative splicing is a key molecular mechanism for increasing the functional diversity of the eukaryotic proteomes. A large body of experimental data implicates aberrant splicing in various human diseases, including cancer. Both mutations in *cis*-acting splicing elements and alterations in the expression and/or activity of splicing regulatory factors drastically affect the splicing profile of many cancer-associated genes. In addition, the splicing profile of several cancer-associated genes is altered in particular types of cancer arguing for a direct role of specific splicing isoforms in tumor progression. Deciphering the mechanisms underlying aberrant splicing in cancer may prove crucial to understand how splicing machinery is controlled and integrated with other cellular processes, in particular transcription and signaling pathways. Moreover, the characterization of splicing deregulation in cancer will lead to a better comprehension of malignant transformation. Cancer-associated alternative splicing variants may be new tools for the diagnosis and classification of cancers and could be the targets for innovative therapeutical interventions based on highly selective splicing correction approaches.

## INTRODUCTION

During evolution the number of genes stopped growing in parallel with the complexity of the proteome. Thus, the human genome contains only 20.000 - 25.000 genes (International Human Genome Sequencing Consortium 2004 [1]), a number not significantly different from that counted in less complex organisms such as sea urchin (23,000) (Sea Urchin Genome Sequencing Consortium 2006) and the nematode worm (19,000) [2]. Moreover, the number of human genes is not sufficient to account for all the proteins revealed by proteomic analysis. How can these paradoxes be explained? Recent cDNA sequencing and microarray data have implicated alternative splicing (AS) as the main source of proteomic and functional diversity in metazoan organisms [3]. Together with alternative promoters and polyadenylation sites, RNA editing and post-translational processing, AS gives rise to an estimated number of at least 100,000 different human proteins.

The term “alternative splicing” describes any situation in which a single primary transcript (pre-mRNA) can be spliced in more than one pattern to generate multiple, distinct mature mRNAs leading to expression of protein isoforms with different structural and functional properties. The "record-holder" for alternative splicing is a Drosophila gene called *Dscam*, with 38,000 splice variants, more than the number of Drosophila genes [4]. In humans at least 70% (and this proportion might be even higher!!) of the genes encode for transcripts that undergo alternative splicing [5], which underscores the importance of this regulatory mechanism in the biology of our species. 

Because of its capacity to generate protein diversity, alternative splicing is expected to play a major role in gene expression regulation, a prediction which is substantiated by the observation that appropriate spatio-temporal generation of splicing variants is involved in many cellular and developmental processes (including sex determination, apoptosis, axon guidance, cell excitation and contraction and many others). It is not surprising, therefore, that deregulation of alternative splicing programs is tightly linked to inherited and acquired human genetic disorders [6]. Indeed, works in the last few years have started to recognize inappropriate alternative splicing as a genetic modifier during tumorigenesis [7]. Many cancer-related genes are regulated by alternative splicing. They encode for proteins involved in all major aspects of cancer cell biology, including cell cycle control, proliferation, differentiation, signal transduction pathways, cell death, angiogenesis, invasion, motility and metastasis [8]. A common signature of cancer cells is a general loss of splicing fidelity, with the concomitant reorganization of splicing profiles, and even switching to specific splicing isoforms usually expressed in other cell types. All these events may contribute to carcinogenesis [8]. Notably, there are several cases of alternative splicing that are restricted to specific cancer types, which clearly involves that particular splicing isoform in tumor progression [8, 9]. Cancer-specific splicing events may be also beneficial to therapy, since they generate novel epitopes against which it is possible to raise antibodies for immunotherapy. Hence, there is great interest in discovering the effect of alternative splicing on the transcriptome complexity in cancer cells and in understanding how this regulatory mechanism contributes to tumorigenesis. This review discusses (1) the basic mechanisms of alternative splicing, (2) the present knowledge on the regulatory mechanisms governing alternative splicing and their deregulation in cancer, (3) the biological consequences deriving from the alteration of splicing in some relevant cancer-related genes and (4) cancer-associated alternative splicing variants with a clear diagnostic/prognostic value that can provide potential therapeutic targets.

## ALTERNATIVE SPLICING

Eukaryotic genomes are characterized by the presence of “interrupted” genes consisting of an ordered succession of coding (exon) and non-coding (intron) sequences. In metazoan, particularly in mammals, interrupted genes account for the vast majority of the genes. Thus, RNA splicing occurs as an obligatory, highly regulated, process. Through pre-mRNA splicing, introns are precisely removed and exons are joined together to reconstitute the reading frame and to generate translatablemRNAs that are then exported to the cytoplasm.

The splicing machinery (called spliceosome), is the largest molecular machine so far described in the cells being composed of five small nuclear ribonucleoproteins (snRNPs U1, U2, U4, U5 and U6) and more than 100 different polypeptides. The spliceosome recognizes short, poorly conserved, *cis*-acting sequence elements at exon–intron boundaries (5’ and 3’ splice sites also known as “donor” and “acceptor” sites) and use them for the cut-and-paste reaction (two sequential trans-esterifications) that removes the intron.

**Fig. (1):**
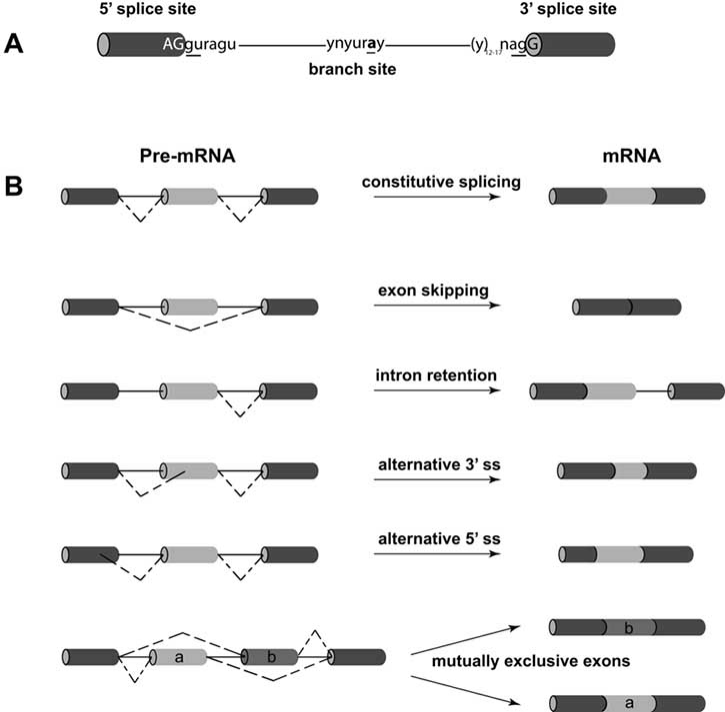
***Cis*-acting sequences required for the splicing reaction and different types of alternative splicing events.** **(A)** Splicing consensus sequences of a typical eukaryotic gene (exon/intron splice site signals, branch site and polypyrimidine tract). **(B)** Alternatively spliced mRNAs result from exon skipping, intron retention, usage of alternative 3’- (acceptor) or 5’- (donor) sites and from selection of mutually exclusive exons. At the protein level, alternative splicing drastically affects the amino acid sequence by deletion or insertion of domains, frame-shifts or stop codons. Alternative splicing in non-coding regions of the mature mRNA might impact on translation and mRNA stability.

By using various combinations of donor and acceptor sites from different exons, through the process of alternative splicing it is possible to produce distinct mRNAs from a single pre-mRNA. Five distinct alternative splicing patterns have been observed: 1) a regulated (cassette) exon, which is sometimes included and sometimes excluded from the mRNA; 2) multiple cassette exons that are mutually exclusive, i.e. the mature mRNA always contains only one of several possible exon choices; 3) in rare cases, a whole intron is retained; 4) alternative donor and 5) acceptor sites can result in exons of different size (see Fig. **1**). Finally, alternative promoters and poly-adenylation sites contribute to the heterogeneity of transcripts encoded by a single gene. In addition to modifying protein features, alternative splicing can affect the stability of transcripts through the nonsense-mediated mRNA decay (NMD) pathway, an mRNA quality-control mechanism which depends on the translation machinery [10]. Recent analyses suggest that approximately 35% of all alternative splicing events in mammalian cells generate mRNA species containing premature termination codons (PTCs), which can be efficiently degraded through NMD [11]. Interestingly, Ni *et al. *[12] have recently shown that exons containing a stop codon are particularly frequent and conserved in genes for splicing factors involved in splicing choices. These exons frequently overlap ultraconserved elements in mammalian genomes.

**Fig. (2):**
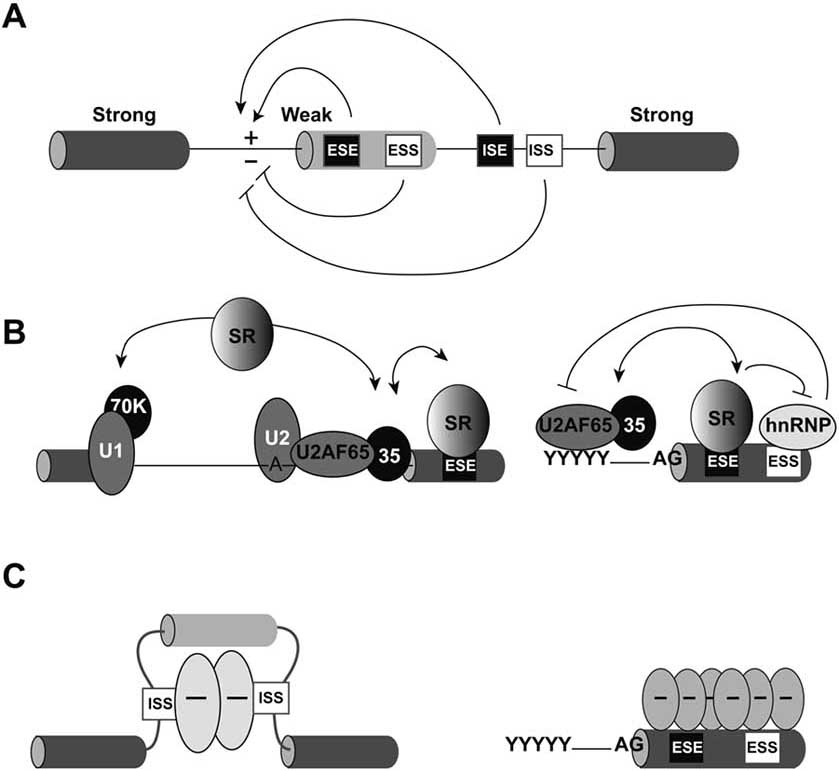
**Cis- and trans-acting regulatory elements that control alternative splicing and models for the function of splicing enhancers and silencers. (A)** Alternatively spliced exons are usually characterized by weak splice sites. Recognition of these sites depends on splicing regulatory elements: exonic splicing enhancers (ESE) and silencers (ESS) and intronic splicing enhancers (ISE) and silencers (ISS). **(B)** ESE elements are bound by splicing factors of the SR family. *Via* interactions with proteins of the splicing apparatus, the RS domain of SR factors promotes the assembly of the splicesome on the adjacent intron (left). In addition, SR factors can counteract the inhibitory activity of hnRNP proteins bound to ESS elements (right). **(C)** The mechanism of action of the silencer elements depend on the their position along the pre-mRNA. In some cases, inhibitory factors (for example hnRNP proteins) bind to ISSs sequences flanking an alternative exon and cause looping out and skipping of this exon (left). Alternatively, inhibitory factors bound to ESSs may polymerize along the exon and displace the ESE-bound SR proteins (right).

From a mechanistic point of view the different types of alternative splicing can be simply viewed as a problem of splice-site recognition by the spliceosome where the decision between inclusion and skipping of a particular exon mainly depends on the recognition and utilization of the splice sites that flank the exon. Alternatively spliced exons are often characterized by short and degenerate splice sites. The intrinsic weakness of these sites, which reflects the reduced affinity for spliceosomal proteins, is the main cause of alternative splicing. The recognition of alternative exons is modulated by an additional layer of information provided by an extensive and complex arrays of auxiliary *cis*-acting elements (non-splice site RNA sequence elements), referred to as enhancers and silencers of splicing that respectively promote and inhibit exon recognition (Fig. **2A**). These are short regulatory elements (~10 nucleotides) that can be found isolated or clustered on the pre-mRNA and are present both within exons (ESEs, Exonic Splicing Enhancers and ESSs, Exonic Splicing Silencers) and introns (ISEs, Intronic Splicing Enhancer and ISSs, Intronic Splicing Silencers) [13]. The best-characterized splicing enhancers are typically purine-rich and function by providing binding sites for serine-arginine (SR) factors, a family (about a dozen) of essential and abundant RNA-binding proteins highly conserved in animal and plant cells [14, 15]. SR factors display multiple roles in constitutive and alternative splicing, as well as in other aspects of gene expression [16]. All members of this family share a modular structure consisting of one or two copies of an N-terminal RNA-recognition motif (RRM) followed by a C-terminal domain of variable length rich in alternating serine - arginine dipeptides (the RS domain). The RRMs determine the RNA-binding specificity, whereas the RS domain mediates specific protein–protein interactions that are essential for the recruitment of the splicing apparatus. However, within the functional spliceosome also the RS domains may directly contact the pre-mRNA. The sequential character of these contacts suggests that RS domain interactions with RNA promote spliceosome assembly [17].

In addition, serine residues of the RS domain are targets of extensive phosphorylation events that influence protein interactions [18], and regulate the activity and sub-cellular distribution of SR proteins [19]. Although several kinases, including SR protein kinases (SRPKs) 1 and 2, CLK/STY, dual-specificity tyrosine-regulated kinase, CRKRS, DNA topoisomerase I, glycogen synthase kinase-3 and AKT, have been shown to phosphorylate SR proteins [19-24], the signal-transduction pathways that regulate alternative splicing are still poorly understood. 

Several models have been proposed for the function of ESEs and SR factors (Fig. **2B**). According to one of these models, ESE-bound SR proteins promote exon definition by directly recruiting the splicing machinery through specific protein-protein interactions mediated by the RS domain [13]. Another model predicts that the main function of ESE-bound SR factors is to antagonize the negative effect on splicing of an inhibitory protein that is bound to a juxtaposed silencer element (ESS) (inhibitor model) [13]. Exon inclusion or skipping is determined by balance of these competing activities, which in turn reflect by relative concentrations of the cognate RNA-binding activator and repressor proteins. These models of splicing enhancement are not necessarily mutually exclusive, as they might reflect different requirements in the context of different exons. 

Splicing silencers identified to date appear remarkably diverse. They may act as binding sites for factors that block access of the splicing machinery to a splice site. Among the proteins interacting with ESSs and ISSs elements there are heterogeneous nuclear ribonucleoproteins (hnRNP), a group of RNA-binding proteins initially recognized as factors that interact with RNA polymerase II transcripts to form hnRNP particles [13]. On two dimensional gels approximately 30 spots were described, called with alphabet letters from hnRNP A1 through U. Similarly to SR factors, hnRNP proteins have a modular structure in which one or more RNA binding domains, generally at the N-terminus, are associated to different “auxiliary” domains. Three types of RNA binding domains (RRMs, hnRNP K homology domain and RGG domain, a protein region rich in Arg-Gly-Gly repetitions) have been identified in hnRNP proteins and shown to provide a certain level of RNA binding specificity [13]. The auxiliary domains are very different in sequence and control the sub-cellular localization and the interaction with other proteins. RNA binding specificity and protein-protein interactions contribute to the assembly of the ribonucleoprotein complexes that are the substrates for the ensuing splicing reaction. The mechanism of hnRNPs action depends on the position of their binding sites along the pre-mRNA. In addition to the inhibitor model described above, hnRNPs bound to ISSs flanking an alternative exon may cause the exon to loop out, resulting in skipping of the exon (Fig. **2C**). Alternatively, inhibitory factors bound to ESS may polymerize along the exon and displace the ESE-bound SR proteins [13, 25] (Fig. **2C**). 

Only in few cases alternatively spliced exons are controlled by tissue-specific splicing regulators [13]. In the vast majority of cases splicing events appear to be controlled by the relative abundance and/or activity of widely expressed antagonistic SR factors and hnRNP proteins through a combinatorial mechanism, with multiple positive and negative factors and sequence elements influencing the final outcome of the splicing reaction. This is exemplified by antagonistic effects on pre-mRNA splicing of SF2/ASF, an SR factor, and hnRNP A1 proteins: high levels of SF2/ASF induce exon inclusion whereas high levels of hnRNP A1 promote exon skipping [26]. Interestingly, the relative expression level of hnRNP A1 and SF2/ASF have been shown to change during neoplastic lung growth [27]. Another example is SRp55 and its antagonistic factor hnRNP I/PTB that control the splicing profile of the *fibroblast growth factor recepto*r (*FGFR1*). Skipping of α exon results in the production of the FGFR1-β isoform which has a higher affinity for fibroblast growth factors [28]. Increased expression of this isoform correlates with cancer in the pancreas and brain and with poor prognosis in breast tumors [29, 30] and its overexpression promotes tumor formation in nude mice [28]. SRp55 binds to a 69-nucleotide ESE and is required for α-exon inclusion while PTB recognizes a sequence upstream of the α-exon and promotes exon skipping and production of FGFR1-β [31].

Recent studies indicate that signaling pathways may control splicing decisions by affecting the sub-cellular distribution and/or activity of splicing regulators. Many SR factors and hnRNP proteins continuously and rapidly shuttle between the nucleus and the cytoplasm [32], which unveils a cytoplasmic function of these proteins, for example in mRNA translation. In this regard, Michlewski *et al*. [33] showed that the splicing factor SF2/ASF stimulates translation initiation by directly recruiting the mammalian target of rapamycin (mTOR) to a subset of mRNAs. Notably, several stress treatments perturb the nucleo-cytoplasmic distribution of some splicing regulators. For instance, exposure of the cells to stress stimuli such as osmotic shock or UVC irradiation, results in a marked cytoplasmic accumulation of hnRNP A1, concomitant with an increase in protein phosphorylation. These effects are mediated by the MKK(3/6)-p38 pathway and correlate with changes in the alternative splicing pattern of an adenovirus E1A pre-mRNA splicing reporter [34]. Moreover, several splicing regulators, including SF2/ASF, are sequestered in nuclear stress bodies after treatments that activate the heat shock response [35]. This capacity to modulate the activity of splicing regulators opens the exciting possibility that stressing conditions, as those in the tumor microenvironment, may influence the splicing profile of a number of genes and thus affect cell identity.

Our knowledge of the molecular mechanisms underlying alternative splicing is still limited. Indeed, there are many exonic and intronic elements for which *trans*-acting mediators remain to be identified and, in most of the cases, regulatory circuits that control splicing decisions and the link with signal-transduction pathways, are not well understood. Deciphering the complex regulatory network underlying alternative splice site choice remains a major challenge in this area of research.

## ABERRANT ALTERNATIVE SPLICING IN CANCER-RELATED GENES

Genome wide analyses indicate that ~75% of human genes encode at least two alternatively spliced isoforms [5]. Thus, alternative splicing seems to be the rule for human genes and it is not surprising that splicing defects in specific genes are causatively linked to genetic disorders. Well-characterized examples are spinal muscular atrophy, myotonic dystrophy, retinitis pigmentosa, Frasier syndrome, hemophilia A, β-thalassemia, atypical cystic fibrosis and some neurodegenerative diseases [6]. It has been calculated that about 15% of point mutations that cause hereditary diseases do alter pre-mRNA processing by affecting canonical 5’ and 3’ splice sites, branch sites or polyadenylation signals [14, 36]. This figure is probably an underestimation since it does not take into account mutations in splicing regulatory elements (enhancers and silencer) that may prevent the interaction with splicing regulators (hnRNPs and SR factors). Indeed, at least 50% of 50 single-base substitutions that cause exon skipping in human genes disrupt splicing enhancers [14, 36, 37]. Frequently, splicing defects due to gene mutations introduce premature stop codons and target the mRNA to degradation by the nonsense-mediated decay pathway (NMD) [10]. The final effect is that, instead of a truncated polypeptide, no protein is produced. 

In the last few years a link has emerged between cancer development and deregulation of alternative splicing [7, 9]. A common feature of cancer cells, in fact, is the general deregulation of splicing, which may lead to the expression of tumor-specific variants and which is most likely promoted by alterations in signaling pathways and by variations in concentration, localization and activity of *trans*-acting splicing regulators [9, 24]. It is still disputed whether these changes in splicing profiles are simply a “noise” occurring in cancer cells or have a direct role in tumorigenesis. As a matter of fact, recent progresses in molecular and cell biology indicate that altered splicing profiles of critical genes may impact on all the major aspects of cancer cell biology [8, 9]. This can be due to inactivation of onco-suppressor [38] or to gain of function of proteins implicated in cancer susceptibility [39] and in tumor progression [8, 9, 40]. 

### *Cis*-Acting Mutations

These are inherited or somatic mutations that affect the splicing process. According to their position and effect on splicing, these mutations can be divided into two subclasses. Subclass I (60% of cases) comprises splicing mutations in the invariant splice-sites and completely abolish exon recognition. These mutations are associated with severe diseases. Subclass II is often associated with a relatively mild phenotype and includes mutations in the variant motifs (such as the alternative splicing poly-pyrimidine tract) and intronic mutations that generate cryptic donor or acceptor sites. An example comes from the tumor suppressor *p53* gene, i.e. the most commonly mutated gene in human cancers. Isoforms of p53 originate through alternative splicing and differ for their tumor suppressor function [41]. Interestingly, “silent” mutations in the p53 gene are predicted to affect splicing because they create splice sites in the middle of an exon [38]. It is worth considering, however, that many mutation screenings focused on exons and ignored non-coding regions [42]. Below, we discuss other examples of mutations that affect splicing of oncogenes and tumor suppressor genes.

Mutations in the APC gene result in familiar adenomatous polyposis (FAP). Recently, it has been reported that an attenuated form of FAP is caused by an insertion of a single T between the second and third position of intron 4 of the APC gene that leads to skipping of exon 4 and to the predicted expression of a truncated protein [43]. Two additional mutations in introns 3 and 4 have the identical effect [44].

Also mutations in splicing regulatory elements, such ESE and ESS sequences, can perturb splicing profiles. A good example is the tumor-suppressor *BRCA1* gene that encodes a protein involved in a variety of cellular processes including transcriptional regulation, recombination, DNA repair [45] and cell apoptosis [46]. Mutations in the *BRCA1* gene are well-known markers of susceptibility to ovarian and breast cancer, the last one being the most common malignancy among women. Mazoyer *et al*. [39] demonstrated that an inherited nonsense mutation within exon 18 disrupts an ESE element (the binding site for the SR factor SF2/ASF) and provokes exon skipping. Computer analysis with the ESEfinder program has identified 23 highly conserved ESEs in the 22 exons of the *BRCA1 *gene [25]. About 60% of these motifs contain nucleotide substitutions reported in the Breast Cancer Information Core [47] suggesting the possibility that targeting of splicing profiles may be the mechanisms through which mutations affect the *BRAC1* function [47]. The BRCA1 gene encodes at least thirty distinct splicing variants that are differentially expressed in tissues [48]. Most of the alternatively spliced transcripts maintain the open reading frame of the original cDNA and have the potential to code for functional proteins. Notably, breast and ovarian cells express a common set of variants suggesting the intriguing possibility that they share some regulatory pathways which instead is absent in leukaemia cell lines that display a different set of isoforms [49]. Many nonsense mutations in the coding region are associated with exon skipping. Thus, a double point mutation in exon 7 of the neurofibromatosis 1 (*NF1*) gene disrupts the consensus binding sites for the SR factors, SC35 and SF2/ASF, and promotes exon skipping. [50]. The *NF1* gene shows one of the highest mutation rate among genes associated with human disorders; simply on the bases of the DNA sequence, it was predicted that 37% of these genomic mutations could determine splicing defects [37]. However, when this prediction was verified through the analysis of the mature mRNAs, it became evident that up to 50% of the NF1 gene mutations are associated with splicing defects. The negative message emerging from this analysis is that we are still unable to infer the splicing profile simply from the primary DNA sequence and that we have to rely on RNA and even protein sequences to understand the effects of any gene mutation [37]. 

Similar observations have been done with a number of other tumor-associated genes such as *BRCA2*, *FHIT*, *KIT*, *MLH1*,* MDM2, MSH2* and *LKB1 *[43, 51-56]. Notably, all these cases, and many others that are continuously described, represent an extension of the concept according to which cancer progression is due to a number of stable genetic mutations that perturb the structure, the function or the abundance of critical proteins. In this sense, splicing defects can be viewed as one of routes through which gene mutations cause tumorigenesis. However, alternative splicing of oncogenes or tumor suppressors could also be affected by mutations in splicing regulators which implies an activity of these factors as oncoproteins or tumor suppressors, depending on their antagonistic functions on splice site selection. For example, the *SRP55* gene (SFR6) is mutated in breast and colorectal cancers [55]. Interestingly, SRp55 controls the splicing profile of several tumor-associated genes, among which *CD44* [57] and *KIT* [58]. Also some hnRNP proteins have been classified as oncogenes. In the 90% of the human myxoid liposarcomas, the t(12;16) translocation generates a fusion between the hnRNP P2 gene, that encoded a multifunctional protein involved in transcription, splicing and mRNA export, and the CHOP gene, encoding for a CCAAT/enhancer binding protein implicated in erythropoiesis, adipocyte differentiation, growth arrest and G1-S cell cycle progression. The product of the hnRNP P2-CHOP fusion contains the amino terminal transcription activation domain of hnRNP P2 and the DNA binding domain of CHOP and its over-expression in nude mice results in tumor formation [59].

### Changes in *Trans*-Acting Factors

More interesting, from our point of view, is the observation, reported by many studies over the last 20 years, that most cancer-associated splicing alterations are not associated with nucleotide changes in the affected genes, implying modifications in the expression and/or activity of splicing regulatory factors [9, 40, 60]. Indeed, changes in the repertoire of SR factors and hnRNP proteins frequently occur in tumors and are accompanied by alterations in the relative abundance of alternative splicing products, a typical signature of cancerous cells with predictable effects on cellular behavior [27, 40, 60, 61]. As a matter of fact, cancerous cell lines show a high level of alternative splicing events that are not conserved between human and mouse and are not expressed in normal tissues [62] strengthening the idea that a change in the level of splicing regulators in cancer cells may severely impinge on gene expression programs. 

A salient example to illustrate how aberrant splicing in cancer can be modulated by altered activity or expression of splicing factors is provided by CD44*, *a trans-membrane glycoprotein involved cell–cell and cell-matrix interactions [63]. Several CD44 isoforms are generated through variable incorporation of 10 alternative exons (v1-v10) in its proximal extra-cellular domain. Standard CD44, lacking all alternative exons, is predominantly express in normal tissues, whereas CD44 isoforms, in particular those containing variant exons v5, v6 and v7, are over-expressed in various tumors and have been implicated in tumor cell invasion and metastasis [63]. The production of different CD44 isoforms correlates with changes in the abundance of SR proteins [61, 64] and several splicing factors (including hnRNPA1, SRp55, SF2/ASF, Tra-2 beta, YB-1 and Sam68) that have been shown to regulate specific variant exons [65-68]. Several lines of evidence indicate that *CD44* splicing is regulated in response to extracellular stimuli. Thus, Matter *et al*. [68] found that the RNA binding protein Sam68 (Src-associated in mitosis 68-kDa) promotes v5 inclusion in response to activation of the Ras-Raf-Mek-Erk pathway. Although mechanistic details are incomplete, it has been suggested that the interaction of phosphorylated Sam68 with exon v5 blocks the repressive activity of hnRNP A1, either by preventing the interaction of hnRNP A1 with the ESS element (by steric hindrance), or by counteracting the inhibitory effect of hnRNP A1 bound to the ESS. Recently, Cheng and Sharp identified SRm160 as another Ras-regulated splicing factor responsible for inclusion of v5 exon in *CD44* transcripts [69]. Importantly, they also showed that silencing of SRm160 decreases cellular invasiveness, linking this splicing regulator to tumorigenesis. 

Another good example of splicing modulation by signaling pathways comes from the *Fibronectin* gene (FN), an extracellular matrix component and a key determinant in controlling proliferation, migration, invasion and metastatic behavior of tumor cells. EDA is a FN splicing isoform generated by inclusion of a single exon (EDA exon, also known as EDI or EIIIA). This splicing isoform is poorly expressed in adult normal tissues, whereas it is present in embryos as well as during wound healing and in certain tumors. Inclusion of the EDA exon is triggered by the activation of the Ras/PI3-kinase/AKT pathway by growth factors. AKT directly phosphorylates the SR proteins 9G8 and SF2/ASF, which in turn bind to the EDA exon and promote its recognition by the splicing apparatus [24]. Activation of the same pathway by insulin regulates the activity of another SR protein, SRp40, and stimulates the inclusion of an alternative exon in the protein kinase C (PKC) II pre-mRNA [70, 71]. Altogether these results support the possibility that deregulation of the Ras/PI3-kinase/AKT pathway, for instance resulting from mutations in its components, could have dramatic consequences on the splicing profile of any pre-mRNAs regulated by 9G8, SF2/ASF, SRp40 and perhaps other SR proteins. An attractive hypothesis is that exons responsive to this signaling pathway belong to a set of genes that function cooperatively to modulate the cell physiology in accordance with the biological role of the signaling molecule. The identification of these exons, therefore, will be of the utmost interest. An indication in favor of this hypothesis comes from the observation that AKT also promotes translation of EDA mRNAs bound by phosphorylated 9G8 and SF2/ASF [24]. Thus, activation of a single signal-transduction pathway controls in an integrated manner both splicing and translation of specific mRNAs and stimulates the production of specific proteins. The final effect is a drastic increase both in the speed and strength of the signaling response as measured by production of the induced protein.

Although these examples provide interesting insights into the effects of signal-transduction pathways on splicing regulation, the molecular characterization is still scanty and clarification of the complete pathway has not yet been provided. Thus, in addition to illustrate a role of signal transduction in splicing control, these cases also suggest the need of further studies to elucidate this important mechanism of gene regulation.

An increasing body of data implicates alternative splicing as a mechanism to control apoptosis or programmed cell death, an essential process in development and in maintenance of cellular homeostasis in multicellular organisms. Bcl-X is a member of the *Bcl*II family that directs mitochondrial breakdown during apoptosis. The usage of alternative 5' splice sites within exon 2 determines the production of two protein isoforms: a long antiapoptotic form (Bcl-XL) and a short apoptosis-promoting protein (Bcl-XS) [72]. Therefore, a shift in the splicing pattern of these transcripts can have deep effects on proliferative activity of cancer cells and on their response to proapoptotic therapies. Several splicing factors, including Sam68, SF2/ASF, hnRNP F/H and SAP155, contribute in controlling the choice between the two alternative 5’-splice sites. In particular, Sam68 over-expression promotes the production of pro-apoptotic Bcl-XS and this effect is reverted upon Sam68 phosphorylation [73]. On the contrary, hnRNP F/H proteins, by binding to a G-rich stretch element, promote the usage of the Bcl-XS - 5’splice site [74]. The ratio of Bcl-X splice variants contribute to determine the sensitivity of the cells to a wide variety of apoptotic agents and may have significance in drugs resistance and chemotherapeutic responsiveness. For example, the lipid ceramide, a mediator/regulator of apoptosis promotes the expression of the pro-apoptotic splicing variants Bcl-XS; the choice between the two alternative 5’-splice sites is controlled by a ceramide responsive element (CRCE 1) located within exon 2 and bound by SAP155 [75]. Moreover, ceramide is able to modulate the phosphorylation status of SR proteins in a phosphatase-1 (PP1)-dependent manner [75]. Interestingly, one of PP1 targets is the SR factor SF2/ASF, another major regulator of Bcl-X pre-mRNAs processing [76]. 

There are several examples of alternative splicing events that control the activity of proteins involved in cell motility and invasion, a pre-requisite for the formation of cancer metastases. This is the case of splicing isoforms of the *androgen *and* estrogen receptors* that are involved in mammary carcinomas [77, 78]. Interestingly, an isoform of estrogen receptor alpha, due to skipping of exon 3 (delta3ER), is a more potent activator of vascular endothelial growth factor than the wild-type receptor [79]. The splice variant Rac1b, which is generated by inclusion of a 57-nucleotide cassette exon, has been shown to lead to anchorage-independent cell growth. Notably, Rac1b is up-regulated in colorectal tumors at various stages of neoplastic progression, as compared to adjacent normal tissues [80]. Other examples, that clearly show the potential functional effect of aberrant splicing on tumorigenesis, are the *fibroblast growth factor receptor 2* (*FGFR2*) [81], the *fibronectin* [82] and the *survivin *[83].

**Fig. (3):**
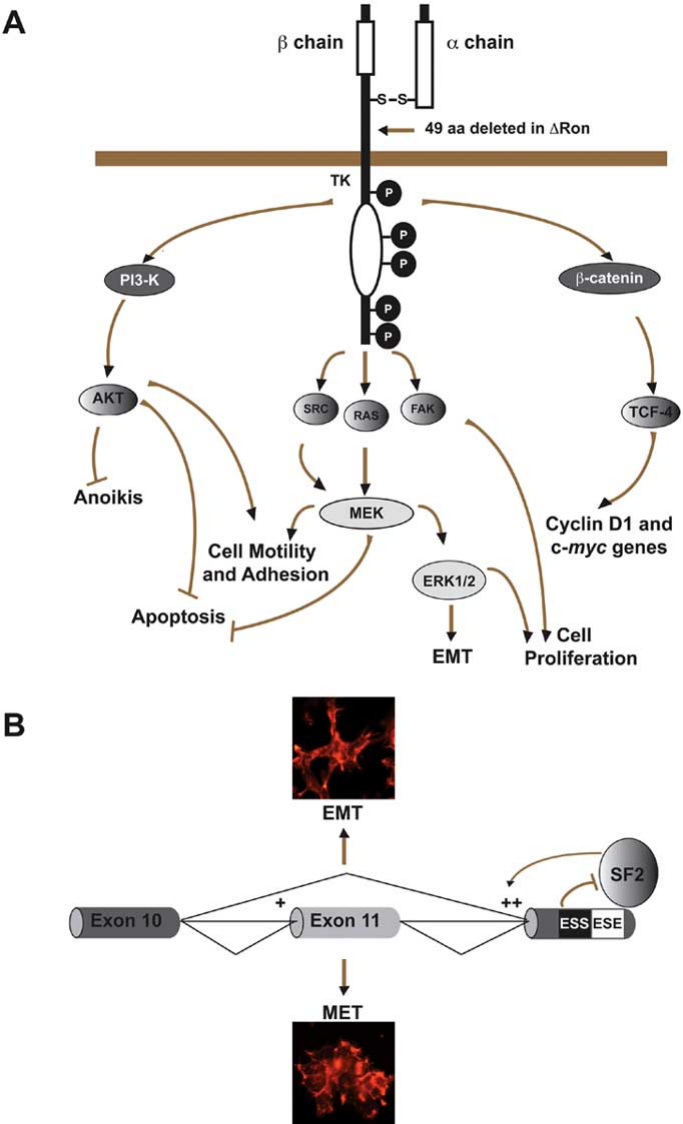
**Schematic representation of the Ron receptor and of the downstream signalling pathways. (A)** Ron is a single-pass, disulfide-linked α/β heterodimer. The α  chain is an extracellular glycoprotein while the β chain is a transmembrane subunit that comprises an extra-cellular sequence, a short trans-membrane segment, a large cytoplasmic portion with intrinsic tyrosine kinase domain (TK) and a C-terminal tail. Residues and domains important for the Ron activity are indicated. In particular, the intracellular domain includes the tyrosine kinase catalytic site (white oval) flanked by distinctive juxtamembrane and carboxy-terminal sequences. Phosphorylation of two tyrosines within the kinase domain positively regulates the enzyme activity, whereas a serine residue in the juxtamembrane domain has a negative regulatory role. Two tyrosine residues in the carboxy-terminal region, when phosphorylated, form a specific docking site for multiple signal transducers and adaptors. Activation of Ron by MSP can initiate signaling through many pathways implicated in tumor progression and metastasis, such adhesion, invasion, mobility, proliferation and inhibition of apoptosis. **(B)** The constitutively active ΔRon isoform is generated through skipping of exon 11. This event is controlled by two adjacent splicing elements, a silencer and an enhancer, located in the central part and at the 3’ end of exon 12, respectively. These two regulatory elements may form a ‘‘control cassette’’ that tunes the strength of the acceptor site of intron 11 and thus the ratio between Ron and ΔRon transcripts. Splicing factor SF2/ASF directly binds to the enhancer and governs its activity. High levels of SF2/ASF increase the strength of the acceptor site of the intron 11, promote the production of ΔRon isoform and trigger morphological and molecular changes typical of the epithelial to mesenchymal transition (EMT). The primary function of the silencer could be to antagonize the enhancer and prevent exon skipping.

Recently, we have used the *Ron* (recepteur d’origine nantais) proto-oncogene as a model to investigate the relationship between alternative splicing and tumor progression [40]. Ron, the human tyrosine kinase receptor for the macrophage-stimulating protein (MSP), is a heterodimeric protein (p185-Ron) composed of α and β subunits both deriving from the processing of a common precursor. Binding to MSP stimulates the intrinsic tyrosine kinase activity of Ron and results in phosphorylation of its docking site for multiple transducer and adaptor proteins leading to the activation of signaling cascades (Fig. **3A**). Along with Met, the hepatocyte growth factor (HGF) receptor, Ron belongs to a subfamily of receptor tyrosine kinases (RTK) with unique expression patterns and biological activities. In addition to promoting cell growth and protection from apoptosis, these receptors control cell dissociation, motility, and invasion of extracellular matrices, a process known as ‘‘invasive growth’’ or ‘‘cell scattering’’ [84]. Invasive growth is physiologically relevant during development, organogenesis and tissue regeneration, but it is also important to mediate invasiveness and to promote malignant progression. Currently, six variants including RonΔ170, Δ165, Δ160, Δ155, Δ110, and Δ55 with various deletions or truncations in the extracellular or intracellular regions have been identified. All these variants are constitutively active but differ in their biochemical and biological properties [for review see [85]]. Moreover, the splicing profile of the Ron gene is frequently altered in epithelial cancers, such as colon and breast cancers, suggesting that the production of multiple Ron isoforms could contribute to pathogenesis of these tumors [40, 86]. Over-expression of any of these isoforms increases cell motility (scatter-like activity). However, only RonΔ160 or RonΔ155 are able to induce focus formation, sustained anchorage-independent growth and the ability to form metastatic tumors in mice [86]. This oncogenic potential is channeled through the PI3-Kinase/AKT pathway [87]. Also Met transcripts undergo alternative splicing and an isoform, called Met-SM, originates from skipping of exon 14 which encodes a 47 amino-acid segment in the juxtamembrane domain. This isoform has been recently shown to play an important role in development and progression of human cancers [88]. Among the mechanisms controlling the expression of the different Ron isoforms in cancer cells, the switch from constitutive to alternative splicing plays the major role. Thus, the elucidation of the regulatory pathways controlling the splicing profile of Ron transcripts will shed new light on both cancer initiation and progression. We have studied in detail the alternative splicing event that leads to the production of Δ*Ron *mRNA. This transcript lacks a 147-bp exon (exon 11). The encoded protein bears a 49-amino-acid in frame deletion in the extracellular domain that affects the proteolytic maturation and results in the accumulation of a single-chain pro-RonΔ165 in the cytoplasm. Moreover, the deleted protein is characterized by aberrant intracellular disulfide bridges that facilitate RonΔ165 oligomerization leading to constitutive phosphorylation and activation [89]. We have shown that the choice between inclusion and skipping of exon 11 is controlled by two adjacent regulatory elements, a silencer and an enhancer, both located in the constitutive exon 12 (Fig. **3B**) [40]. The binding of the SR protein SF2/ASF to ESE stimulates skipping of exon 11 and the production of ΔRon. Thus, overexpression of SF2/ASF in cells that normally express Ron triggers the production of the RonΔ165 with dramatic consequences on the cell properties. Indeed, similarly to what observed after ΔRon over-expression, an increased expression of SF2/ASF profoundly affects cell morphology and triggers nuclear accumulation of β-catenin, reorganization of actin cytoskeleton, and down-regulation of E-cadherin, a tumor and invasion suppressor in human carcinomas. All these morphological and molecular changes represent hallmarks of the epithelial to mesenchymal transition (EMT), which is implicated in the metastatic spreading of human carcinomas (Fig. **3B**) [90]. Notably, knockdown of SF2/ASF by RNA interference (RNAi) reduces the levels of ΔRon and concomitantly decreases cell motility. Similarly, RNAi specifically directed against *ΔRon* reduces cell motility and partially reverts the morphological changes induced by SF2/ASF over-expression.

Given the reported up-regulation of several SR proteins, including SF2/ASF, during tumor progression [27, 60], it is tempting to speculate that splicing factor SF2/ASF could promote the malignant transformation by inducing a ΔRon-mediated EMT. This hypothesis is consistent with a recent report showing that SF2/ASF behaves as a *bona fide* protooncogene [60]. SF2/ASF is up-regulated in a set of human tumors and, the gene coding for SF2/ASF is specifically amplified in some breast tumors but not in normal breast tissue from the same patient. Limited over-expression of SF2/ASF, comparable to that in cancer samples, resulted in tumor formation in nude mice. Notably, several endogenous splicing targets of SF2/ASF, among them a novel oncogenic isoform of the mTOR substrate, S6K1, are essential for SF2/ASF-mediated transformation. In addition, RNA interfering (RNAi) of SF2/ASF or the oncogenic S6K1 isoform, resulted in reversion of the transformed phenotype [60].

The identification of splicing regulators as a novel class of proto-oncogenes may unveil novel aspects of cancer biology and offer new opportunities for diagnosis and therapy.

Although all the examples reported above unquestionably prove that changes in the splicing activity of tumor cells is linked tumor progression, yet the molecular mechanisms responsible for these changes remain undefined. Several questions still deserve investigation. Which are the events that lead to deregulation of expression and/or activity of members of SR and hnRNP families? Is there a generalized reorganization of the splicing machinery in cancers cells or does the splicing switch result from a deregulation of a subset of splicing regulators? How do signal transduction pathways cooperate to affect splicing profiles to determine malignant conversion?

### Alternative Splicing Signatures as Potential Diagnostic/Prognostic Indicators and Prospect for Therapies

In the previous sections we have discussed several examples that illustrate a causative link between alternative splicing and development of neoplasia. It is worth noticing that in many cases splicing isoforms appear to be cancer-specific [8]. Moreover, there are evidences that in different tissues a different set of genes undergoes changes of splicing profiles during tumorigenesis [91]. Thus, in kidney tumors, *Tropomyosin 1*, *Actinin α 1*, *Integrin β 4*, *Catenin δ 1* and *Fibroblast growth factor receptor 2 *change their alternative splicing patterns, whereas *N-glycanase1 *does not, despite its apparent alterations in lung and uterus tumors. These findings argue against the idea that changes in splicing profiles may result from a generalized alteration of splicing machinery in cancers cells and suggest that only a specific set of splicing regulators are affected by the tumorigenesis in a tissue-specific manner. The identification of cancer-specific, tissue-specific alternatively spliced isoforms immediately incited their potential use as diagnostic, prognostic, or predictive biomarkers [8]. Although large-scale studies have yet to be carried out, initial results are promising. An important correlation between aberrant alternative splicing and tumor progression has been shown for *CD44* [63]. In particular, the isoforms containing the variant exon v6 are frequently up-regulated in head and neck carcinomas or in advanced stage gastric cancers and *CD44v6* positive tumors are associated to poor prognosis [92]. Also, aberrant alternative splicing of *MDM2* transcripts were found to associate with a prognostic factor for poor survival in patients with breast cancer [93], whereas *HDMX *has been linked to soft-tissue sarcoma [94]. Furthermore, altered expression of a spliced variant of *p73*, a p53-related protein, seems to be a negative prognostic marker in patients with neuroblastoma [95].

During the past decade classical gene expression profiling performed by microarray has been a powerful tool for the discovery of new cancer biomarkers. However, most array platforms developed to date are not designed to distinguish mRNA isoforms. An important direction for the future will be the application of genome-wide screens designed for the analysis of alternative splicing signatures associates with cancer but virtually absent in normal cells. These splicing-sensitive microarrays have the potential to better identify tumor sub-classes. Moreover, they do not require that cause–effect relationships between splicing profiles and cancers are identified (or even that they existed!!), but only that this associations are sufficiently consistent to be predictive. Interestingly, Li *et al*. [96] have used an approach to simultaneously quantify changes in splicing (“splicing switch”) and transcript abundance (up- and down-regulation). The advantage of this “two-dimensional’’ profiling strategy is that it works with partially degraded biological samples such as RNA derived from tissue blocks that have been formalin-fixed and paraffin-embedded. These authors identified a specific set of mRNA isoform biomarkers for prostate cancer using independent panels of tissue samples. In particular, they reported two variants of *AMACR* gene (which encodes a-methylacyl-CoA racemase) resulting from the alternative use of the last exon coupled with alternative polyadenylation: one isoform showed a quantitative up-regulation in prostate cancer compared with normal prostatic tissues, the other seemed to be expressed only in prostate cancer. More recently, novel microarray technology has been used to measure whole-genome exon expression in 102 normal and cancer tissue samples of different stages from colon, urinary bladder, and prostate [97]. Seven genes with tumor-specific splice variants were identified (*Alpha-actinin 1*, *Caldesmon*, *Collagen, type VI alpha 3*, *Leucine-rich repeat interacting protein 2*, *Phosphatidylinositol-4 kinase catalytic beta polypeptide*, *Tropomyosin 1* and *Vinculin*). Notably, tumor-specific splicing patterns of *Alpha-actinin 1*, *caldesmon *and *Vinculin*, key cytoskeleton components, were found in all three organs suggesting that they may represent general cancer related splicing events. Moreover, in silico protein analysis predicts that these identified cancer-specific splice variants encode proteins with potentially altered functions, indicating that they may be involved in pathogenesis and hence represent novel potential biomarkers and drug targets [97]. 

**Fig. (4):**
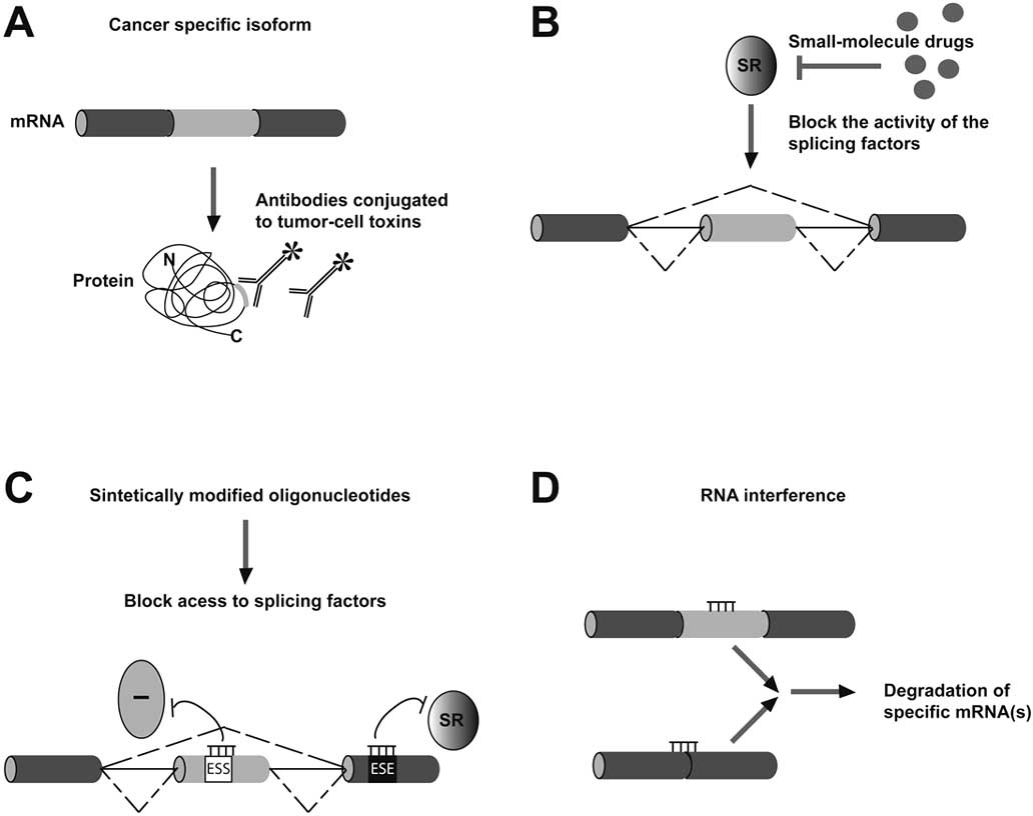
**Potential use of therapeutic approaches that target alternative splicing.** Various strategies are currently being used to exploit alternative splicing for treatment of cancer. **(A)** In many cases, cancer-restricted splice variants contain unique epitopes that could be used as the targets for specific antibodies conjugated to tumor-cell toxins. **(B)** Many chemical compounds have been found to affect splicing of numerous genes. Although the mechanisms by which splicing patterns are altered are still poorly understood, several compounds are able to block the activity of SR-protein kinases (SRpKs) and, consequently, reduce the phosphorylation state of SR splicing factors. **(C)** Synthetically modified oligonucleotides, such as phosphorothioate, morpholino phosphorodiamide and 2´-O-methylethyl, are able to block the interaction of the spliceosome machinery with a specific site. Moreover, they are more stable and active than regular nucleotide backbones and display low toxicity *in vivo.* **(D)** Cancer-specific mRNA transcripts that contain unique sequences can be targeted using RNAi mediated degradation.

Cancer-specific splice variants may not only serve as diagnostic and prognostic tumor markers, but also provide potential targets for the development of new therapeutic strategies (Fig. **4**). One promising avenue towards the development of more selective anticancer drugs consists in targeted delivery of bioactive compounds to the tumor by means of molecules (for example antibodies) that are specific for tumor-associated markers. The accessibility as well as the restricted pattern of expression of the antigene is an important criterion in the selection of a target for bio-molecular intervention. Notably, many receptors mediating cell-cell and cell-matrix interactions are regulated by alternative splicing and specific alternatively spliced variants of these molecules are associated with many human malignancies [8, 9]. Interestingly, nearly 90% of alternative splicing events affect regions located on the exposed surface of protein [98] and for many cell membrane molecules, alternative exons encode novel epitopes that are usually found in the extracellular domain of the protein [8, 9]. These epitopes seem to be ideally suited for tumor-targeting strategies. Once more, the CD44 protein, discussed in detail in the previous section, provides a remarkable example since radiolabeled antibodies directed against the CD44-v6 isoform appear to offer a promising therapeutic tool and are currently in clinical trials for treatment of head and neck cancer [99]. However, despite these promising developments, solid tumors are frequently relatively resistant to antibody-based therapies [100]. This is due, in part, to the relative inaccessibility of tumor cells and to the poor penetration of antibodies into the tumor tissue. Since tumor cells are separated from the blood by endothelial cells and extracellular matrix components surrounding the vasculature, tumor uptake is highly limited by the antibody’s ability to cross this layer. Importantly, therapeutic approaches directed against tumor neo-vasculature have gained significant for a number of reasons: *(i) *tumor vascular endothelial cells are readily accessible for drugs *via *the blood circulation; *(ii) *there is growing evidence that inhibition or regression of tumor vessels leads to cancer cell death since they rely on blood vessels for nutrients and oxygen to satisfy their metabolic needs [101]. An interesting example is the EDB alternatively spliced isoform of Fibronectin (FN). The EDB isoform is generated through inclusion of a single exon encoding a 91 amino acids region and it is present in neoplastic tissue blood vessels while it is virtually absent in mature/adult tissues. EDB is involved in the regulation of endothelial cell proliferation and vascular morphogenesis; an EDB–specific radiolabeled antibody is currently on phase II clinical trials for anti-angiogenic cancer treatment [102]. Recently, it has been shown that the alternatively spliced extra-domain A (EDA) of fibronectin may represent another, equally attractive, target for the antibody-based delivery of bioactive agents to the neovasculature not only of solid tumors, but also of metastatic lesions [103]. Similarly, splicing variants could represent novel leukemia-specific antigens with potential use in immunotherapeutic approaches. In Ph-positive leukemias, the t(9;22) translocation generates the Bcr/Abl fusion proteins, whose constitutive tyrosine kinase activation is responsible for the appearance of the leukemia phenotype [104]. In addition to the main *BCR/ABL* fusion transcripts, it has been reported that *BCR/ABL* transcripts, arising from alternative splicing, are also produced in a high percentage of chronic myelogenous leukemia (CML) and acute lymphoblastic leukemia (ALL) patients [105]. In particular, *BCR/ABL* alternative transcripts involving ABL exon 4 are very attractive because the resulting fusion proteins contain a novel immunogenic epitope that is able to elicit an antigen-specific T-cell response [105]. 

Innovative therapeutic approaches could also be designed to target the splicing machinery. For example, post-translational modifications of splicing regulators may be relevant for tumor progression. SRPK1 is up-regulated in breast and colonic tumors compared with adjacent normal epithelium, and the levels of the kinase increase along with tumor grade [106]. Strong expression of SRPK1 protein was also evident in the majority of breast and colonic tumor cell lines [106]. Interestingly, down-regulation of SRPK1 using small interfering RNA (siRNA) affects the expression of key apoptotic factors BAX and BCL2, increases the proportion of the tumor cells (but not of nontrasformed cells) undergoing apoptosis and amplifies their sensitivity to two commonly used chemotherapeutic agents such as gemcitabine and cisplatin. [106, 107]. These results suggest that pharmacological inhibition of SRPK1 activity may be effective as stand-alone agent or in combination with conventional chemotherapeutic regimens. Moreover, small molecules with SRPK-blocking activity, such as Clk1/Sty inhibitors [108] or potent inhibitors of the splicing reaction, such as indole derivatives [109], can be used to alter splicing patterns. Interestingly, recent data showed that the spliceosomal protein E (SmE) functions as suppressor of tumor cell growth in p53-independent manner [110]. Thus, considering that a large portion of human cancers are defective in p53 activity, targeting SmE in cancer gene therapy will be of particular interest.

Different therapeutic approaches are currently being explored to correct aberrant alternative splicing at the RNA level. These strategies can either alter alternative splicing patterns of specific genes or recognize particular mRNA species to elicit their degradation (for example, RNAi, microRNA and ribozymes). Correction of splicing defects through manipulation of mRNA requires the use of gene-therapy strategies, which are often connected with problems such as delivery, toxicity and immunogenicity. SiRNA-based therapy has shown great promise for many diseases such as cancer. Major targets for siRNA therapy include oncogenes and genes that are involved in angiogenesis, metastasis, cell survival, antiapoptosis and resistance to chemotherapy [111]. Moreover, the latest generations of antisense oligonucleotides that contain chemical modifications appear more stable compared to conventional nucleotide backbones. Some of these oligos prevent ribosomal assembly, and hence mRNA translation, and seem to be well tolerated in patients [112]. In addition, synthetically modified oligonucleotides targeted through sequence specific hybridization to splice sites or adjacent sequences may block inappropriate exon selection by inhibiting the binding of *trans*-acting factors. They have been successfully applied to correct aberrant splicing of *β-globin*, *CFTR*, *dystrophin*, *tau* genes [113] and to repress the generation of cancer-related splice variants of *FGFR1*, *Bcl-X*, and *MDM2. *Alone or in combination with chemotherapeutic agents, such as cisplatin, irinotecan, paclitaxel, fluorouracil they may proficiently used in therapy [114]. Finally, we have designed a phosphorodiamidate morpholino oligomer targeting the region of the *Ron* enhancer containing the SF2/ASF binding site (Mo-SF2) to sterically block the binding of this splicing factor and, thus, the skipping of exon 11. The efficacy of Mo-SF2 was tested using human gastric carcinoma KATOIII cells characterized by high levels of SF2/ASF, ΔRon and invasive phenotype. We have found that Mo-SF2 inhibits *ΔRon* splicing and corrects the cellular morphology and the migration properties of the cells [unpublished data].

## CONCLUDING REMARKS

Following the sequencing of entire eukaryotic genomes, alternative splicing has emerged as the strategy dedicated to the interpretation of the genetic information. As widely discussed in this review, in fact, this mechanism of pre-mRNA processing accounts for a large proportion of the proteomic diversity in higher eukaryotes. While transcription regulation determines the presence and abundance of primary gene transcripts, alternative splicing is designed to manipulate the message in response to a number of stimuli. In this way it may tune protein features, such as the ability to participate to hetero-complexes or the sub-cellular localization, in order to meet the needs of the cells. Rarely are alternative splicing programs associated to “yes or no” decisions; in most of the cases splicing products co-exist in a single cell and contribute to the cell ability to cope with a number of different internal and external stimuli. From this viewpoint, therefore, this mechanism appears to be intimately connected to the complexity of metazoan organisms which, once more, underscores the necessity to understand the combinatorial rules underlying splice site choices as an obligatory step to reach a comprehensive picture of the logic of living matter.

Deciphering the biological implications of this regulatory process is a challenging task that requires the integration of a large body of data relative to the *cis*-acting sequences and *trans*-acting factors involved, the regulatory circuits and signaling pathways that modulate splice site decisions and finally the physiological and/or pathological consequences resulting from the expression of the various splicing isoforms. The attainment of this result will be certainly hastened by the recent development of new and highly sophisticated bio-informatics and system biology approaches.

An important breakthrough to fully understand how alternative splicing permeates the expression programs is expected to derive from the application of high-throughput methodologies. During the last decade, biomedical sciences have been strongly influenced by “omics”: genomics, proteomics, transcriptomic and metabolomic. The application of high-throughput approaches to study splicing profiles is just at its beginning. This delay is certainly due to a number of technical problems deriving from the necessity to contemporary assess for each gene the absolute level of total transcripts and the relative abundance of any splicing isoforms. Moreover, the interpretation is significantly complicated by the necessity to understand the physiological implications resulting from a change in splicing profiles. 

However, it is easy to predict that this type of analysis will provide crucial information to unveil the regulatory pathways underlying co-regulation of splicing profiles and to understand the relevance of alternative splicing in the context of the organism development. The hope is that splicing-sensitive arrays will guide the identification of circuits that, similarly to signal transduction and transcription pathways, may be causatively linked to development programs, organogenesis, body plan definition and cell identity. Another open question concerns the identification of the sub-genome that does not undergo alternative splicing events. Do these genes identify any particular critical cellular function?

Thus, the change of perspective, from detailed characterization of molecular mechanisms to global approaches, it is expected to improve our understanding of the physiological relevance of alternative splicing and to drastically increase the comprehension of important physiological and pathological conditions such as the neuronal plasticity and the complexity of cancer. We are confident that system biology approaches can help the identification of alternative splicing events that may play a critical role in tumor progression. This will offer the opportunity to develop innovative strategies for therapeutical intervention that target specific alternative splicing variants.
